# *miR-325-3p* Reduces Proliferation and Promotes Apoptosis of Gastric Cancer Cells by Inhibiting Human Antigen R

**DOI:** 10.1155/2023/6882851

**Published:** 2023-09-19

**Authors:** Zhengwei Huang, Yacan Luo, Congcong Chen, Chaoyang Zhou, Zhengkang Su, Chang Cai, Xi Li, Wenzhi Wu

**Affiliations:** ^1^Department of Gastroenterology and Hepatology, The First Affiliated Hospital of Wenzhou Medical University, Wenzhou, Zhejiang, China; ^2^The Affiliated Kangning Hospital of Wenzhou Medical University, Zhejiang Provincial Clinical Research Center for Mental Disorder, Wenzhou, China; ^3^Intensive Care Unit, The People's Hospital of Yuhuan, Yuhuan, China

## Abstract

Human antigen R (HuR), also known as ELAVL1, is a widely expressed RNA-binding protein (RBP) that has a significant impact on the development and advancement of tumors. Our previous study found that 5-fluorouracil (5-FU) may impede the proliferation and increase apoptosis in gastric cancer cells by reducing the nucleocytoplasmic shuttling of HuR. However, how posttranscriptional regulation influences HuR functions in gastric cancer remains to be elucidated. Here, we demonstrated that *miR-325-3p* has the potential to regulate the expression level of HuR by directly binding to its 3′UTR, which in turn led to a significant reduction in proliferation and an increase in apoptosis in gastric cancer cells. In addition, xenograft experiment showed that knockdown of HuR or overexpression of *miR-325-3p* group exhibited smaller tumor sizes after transplant of gastric cancer cells into zebrafish larvae. Thus, our findings offer new insights into the pathogenesis of gastric cancer and may potentially assist in identifying novel targets for drug therapy.

## 1. Introduction

Patients with gastric cancer have a high mortality rate and a poor prognosis [1, 2]. As of 2020, gastric cancer is the fifth most commonly diagnosed cancer and the fourth leading cause of cancer-related deaths [3]. The primary treatment for gastric cancer is surgery [4]. Despite undergoing gastrectomy, patients diagnosed with gastric cancer have a 5-year survival rate of only 41.6% [5]. To date, gastric cancer remains a significant threat to human life globally. Thus, comprehending the fundamental molecular mechanisms of gastric cancer development may offer an extensive range for discovering new drugs.

HuR binds to mRNAs 3′UTR and is extensively involved in posttranscriptional gene regulation as an RNA-binding protein [6]. Throughout the human body, HuR is ubiquitously expressed due to its membership of the embryonic lethal abnormal vision-like (ELAVL) family. HuR can regulate the expression of various proto-oncogenes, cytokines, and growth factors which have confirmed roles in the growth, invasion, and metastasis of multiple types of tumors including colorectal cancer [7], cervical cancer [8], gastric cancer [9–11], and other cancers [12–14]. Inhibition of HuR expression has been shown to reduce tumor cell proliferation and increase cell apoptosis [10, 15, 16]. Recently, HuR has emerged as a promising target for cancer therapy [12]. Various pieces of evidence point out that HuR, modulating drugs with low and manageable side effects, will be a future direction for cancer therapeutics [17, 18].

MicroRNAs, approximately 18–25 nucleotides in length, are a class of small noncoding RNAs that are widely expressed *in vivo* and involved in many life activities [19]. Abnormal expression of miRNAs have been related to multiple diseases and may even serve as biomarkers for malignant tumors [20, 21]. For example, *miR-22* has been reported to be involved in regulating HuR and participating in the development of colorectal cancer [7]. *miR-519 *delayed cell proliferation by decreasing the level of RNA-binding protein HuR [8] and hampers the progression of gastric cancer by targeting HuR [11]. Certain miRNAs, such as *miR-34* analogs and anti-miRs targeted at *miR-122*, have been included in clinical trials as potential therapeutic targets [22]. Meanwhile, the expression level of *miR-325-3p* is significantly reduced in gastric cancer [23]. In addition, *miR-325-3p* also plays a role in regulating resistance to chemotherapy and immunotherapy [24–26]. These findings suggest a potential association between the expression of *miR-325-3p* and the development of gastric cancer.

The aim of this study is to investigate the role of *miR-325-3p* in regulating post-HuR transcription in gastric cancer. This study hypothesizes that *miR-325-3p* regulates intracellular expression of HuR by binding to its 3′UTR, which affects the proliferation and apoptosis of gastric cancer cells. The hypothesis was further validated by luciferase activity assay, TUNEL assay, cell Viability assay in vitro and xenograft model in zebrafish.

## 2. Materials and Methods

### 2.1. Animals

The zebrafish (*Danio rerio*) were obtained from the National Zebrafish Resource Center of China (Wuhan, China) and raised according to the guidelines provided in *The Zebrafish Book* [27]. For mating, two males were placed with one female in a tank separated by a baffle plate. The larvae were reared at a temperature of 28.5°C in an E3 medium and fed three times a day with *Artemia nauplii*. All animal experiments were carried out by the guidelines for animal care and approved by the Ethical Commission of Wenzhou Medical University.

### 2.2. Cell Culture

SGC-7901 and HGC-27 cells were purchased from the Shanghai Institute of Biochemistry and Cell Biology (Shanghai, China) and cultured in an RPMI-1640 growth medium containing 10% fetal bovine serum (FBS) in a 5% CO_2_ humidified incubator at 37°C.

### 2.3. Transient Transfection

Transfection was performed using the transient transfection method according to the Lipofectamine™ 3000 Reagent procedure (L3000001, Thermo Fisher Scientific, USA). The ratio of plasmid to transfection reagent in one well of a 6-well plate was 75 pmol: 1 *μ*L in 250 *µ*L Opti-MEM™ medium according to the supplier's instructions. The mixture was added to each well together with 2 mL of the medium. The sequences of siRNA for *HuR* are as follows (3′-5′): sense1: *UACCAGUUUCAAUGGUCAUAA*, antisense1: *UUAUGACCAUUGAAACUGGUA* (siHuR1); sense2: *GCGUUUAUCCGGUUUGACATT*, antisense2: *UGUCAAACCGGAUAAACGCTT* (siHuR2). The negative control sequence of siRNA (siNC) is as follows: sense:*UUCUCCGAACGUGUCACGUTT*, antisense:*ACGUGACACGUUCGGAGAATT*. miRNA-negative control (miR-NC), *hsa-miR-325-3p* mimics, *hsa-miR-325-3p* inhibitor, siNC, siHuR1, and siHuR2 were synthesized and purified by GenePharma (Shanghai, China).

### 2.4. Western Blot Analysis

Western blot analyses were performed as described in a previously published paper [28]. Total proteins were extracted from SGC-7901 and HGC-27 cells 48 hours after transfection by the radioimmunoprecipitation assay (RIPA) lysis buffer containing protease inhibitor cocktail (PIC), and the protein concentration was measured using the bicinchoninic acid (BCA) assay (Beyotime, Shanghai, China). The protein samples were separated by 12% sodium dodecyl sulfate-polyacrylamide gel electrophoresis (SDS-PAGE) and transferred to polyvinylidene difluoride (PVDF) membranes (Millipore, MA, U.S.A.). Membranes were blocked with 5% milk and incubated with the following primary antibodies: rabbit anti-BAX (catalog# 50599-2-Ig, 1 : 1,000; Proteintech, Wuhan, China), mouse anti-Caspase3 (catalog # 66470-2-Ig, 1 : 1,000; Proteintech, Wuhan, China), mouse anti-HuR (catalog# sc-5261, 1 : 1,000; Santa Cruz, Dallas, U.S.A.), and mouse anti-VINCULIN (catalog# ab129002, 1 : 1,000; Abcam, Cambridge, UK), and then incubated with HRP-conjugated goat anti-rabbit or goat anti-mouse secondary antibodies (catalog# 1460 or 31430, both diluted to 1 : 10,000; Thermo, Waltham, U.S.A.) for 1 hour at room temperature (RT). Finally, protein bands were detected by chemiluminescence (ECL) reagents. The intensity of the bands was quantified using ImageJ software (National Institutes of Health, Maryland, U.S.A).

### 2.5. Quantitative Real-Time Polymerase Chain Reaction (qRT-PCR)

Total RNA and microRNA were extracted from SGC-7901 and HGC-27 cells (48 hours after transfection) by using the TRIzol reagent (Sigma-Aldrich, St Louis, Missouri) or RNAiso for small RNA reagent (Takara, Dalian, China) and then reverse-transcribed into cDNA with PrimeScript™ RT Master Mix (Takara, Dalian, China) or Mir-X miRNA First-Strand Synthesis Kit (Takara, Dalian, China). cDNA was used as the template for gene quantification by qPCR using SYBR® Premix Ex Taq™ (Takara, Dalian, China) according to the manufacturer's protocol. The primer sequences used are as follows (5′-3′): *HuR*-F: GTCCTCGTGGATCAGACTAC;* HuR*-R: TCATGTGATCGACGCCCATG; *BAX*-F: CGGGTTGTCGCCCTTTTCTA;* BAX*-R: GGAGACAGGGACATCAGTCG;* BCL2*-F: CTTTGAGTTCGGTGGGGTCA; *BCL2*-R: GGGCCGTACAGTTCCACAAA; *CASPASE3*-F: ATGGAGAACAACAAAACCTCAGT;* CASPASE3*-R: TTGCTCCCATGTATGGTCTTTAC;* CASPASE8*-F: TGCTTGGACTACATCCCACAC;* CASPASE8*-R: TGCAGTCTAGGAAGTTGACCA;* CASPASE9*-F: TCCTGGTACATCGAGACCTTG;* CASPASE9*-R: AAGTCCCTTTCGCAGAAACAG;* RPLP0*-F: GTCCAACTACTTCCTCAAGATCATCCA;* RPLP0*-R: ACATGCGGATCTGCTGCAT. *RPLP0* was used as the internal control.

### 2.6. TUNEL Assay

Apoptotic cells were measured via TUNEL staining, using in situ cell death detection kit (Roche, Basel, Switzerland) as described by Lv et al. [29]. SGC-7901 and HGC-27 cells were fixed at room temperature for 30 minutes using 4% paraformaldehyde (PFA) solution and then treated with permeation solution (0.1% Triton X-100) for 5 minutes at 4°C. Following washing with phosphate-buffered saline (PBS), the cells were incubated with TUNEL reagent, which contained 10% terminal deoxynucleotidyl transferase and 2% fluorescent isothiocyanate-dUTP, for 1 hour at 37°C. Subsequently, the cellular nucleus was detected by staining the cells with diamidino-2-phenylindole (DAPI) for 30 minutes at room temperature. Finally, the cells were sequestered using an antifluorescence quencher, and the number of apoptotic bodies and TUNEL-positive SGC-7901 and HGC-27 cells were determined with fluorescence microscope.

### 2.7. Cell Viability Assay

Cell viability was determined with the Cell Counting Kit 8 (CCK-8, Beyotime, China) assay. SGC-7901 and HGC-7901 cells were seeded in 96-well plates at a density of 2,000 cells per well. Then, various vectors were transfected into the cells as needed for the experiment. At indicated time points, 10 *μ*L of the CCK-8 solution was added to each well of the plate and then incubated for another hour before measuring the absorption intensity of each well at 450 nm.

### 2.8. Dual-Luciferase Reporter Assay

Two conserved binding sites were identified in the 3′UTR of HuR using the TargetScan database (https://www.targetscan.org/mamm_31/). The 3′untranslated region (UTR) of *HuR* was cloned into the pmirGLO luciferase reporter vector, containing the predicted binding sites.

*miR-325-3p* mimics or miR-NC and *HuR* 3′UTR WT or *HuR* 3′UTR MUT vectors were cotransfected into SGC-7901 and HGC-27 cells. After 48 hours of transfection, the luciferase activity was determined using dual-luciferase reporter analysis system.

### 2.9. Tumor Xenograft Model in Zebrafish Larvae

SGC-7901 cells were transfected with the PEGFP-N1 plasmid and selected with G418 to establish gastric cancer cell lines that stably express green fluorescent proteins (GFP). SGC-7901 cells expressing GFP were transfected with siNC, siHuR, miR-NC, and *miR-325-3p* mimics or inhibitor. Transfected cells were then injected into the yolk sac of zebrafish larvae at a 2-day postfertilization stage (200 cells/fish) [30]. Tumor growth was monitored 24 hours later via fluorescence microscope (EVOS FL Auto Cell Imaging System, Thermo).

### 2.10. Statistical Analysis

GraphPad Prism 9.3.1 statistical software was used for data analysis. The mean ± S.E.M. (standard error of the mean) was presented for all data. The normal distribution of all datasets was verified using the Kolmogorov–Smirnov test. To determine the statistical significance, unpaired two-tailed *t*-tests or ANOVA tests followed by Dunnett's multiple comparisons test were used as appropriate. Significance was defined as ^*∗*^*P* < 0.05, ^*∗∗*^*P* < 0.01, ^*∗∗∗*^*P* < 0.001, and ^*∗∗∗∗*^*P* < 0.0001.

## 3. Results

### 3.1. Knockdown of HuR Inhibited Proliferation and Promoted Apoptosis in Gastric Cancer Cells

**Figure 1:**
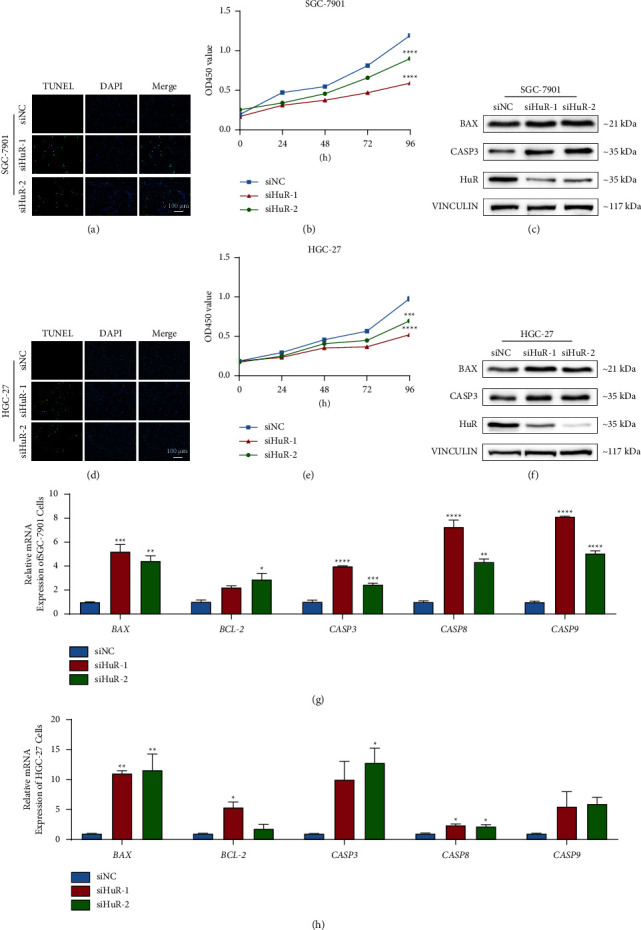
Knockdown of HuR inhibited proliferation and promoted apoptosis in SGC-7901 and HGC-27 cells. (a, d) Detecting the apoptosis cells of SGC-7901 and HGC-27 by TUNEL assay after HuR knockdown. (b, e) Evaluating the proliferation by CCK-8 assay after HuR knockdown within 96 hours. (c, f) Protein levels of BAX, Caspase 3 and HuR by Western blot. (g, h) Apoptosis-related gene expression levels by qRT-PCR after HuR knockdown (^*∗*^*P* < 0.05, ^*∗∗*^*P* < 0.01, ^*∗∗∗*^*P* < 0.001, and ^*∗∗∗∗*^*P* < 0.0001, compared to siNC group).

To investigate the effect of HuR on apoptosis and proliferation in gastric cancer cells, siRNA was transfected to knockdown HuR in SGC-7901 and HGC-27 cells. The proportion of apoptotic cells was significantly higher in knockdown of HuR group (Figures 1(a) and 1(d)) than that of siNC group. Supression of HuR upregulated the protein levels of BAX and Caspase3 (CASP3) (Figures 1(c) and 1(f)), and mRNA expressions of *BAX*, *caspase3*, *caspase8*, and *caspase9* in gastric cancer cells (Figures 1(g) and 1(h)). In addition, cell growth rate of the siHuR group was remarkably lower than that of the siNC group (Figures 1(b) and 1(e)). In summary, knockdown of HuR blocks cell proliferation and promotes apoptosis in gastric cancer cells.

### 3.2. Overexpression of *miR-325-3p* Blocked Expression Levels of *HuR*

**Figure 2:**
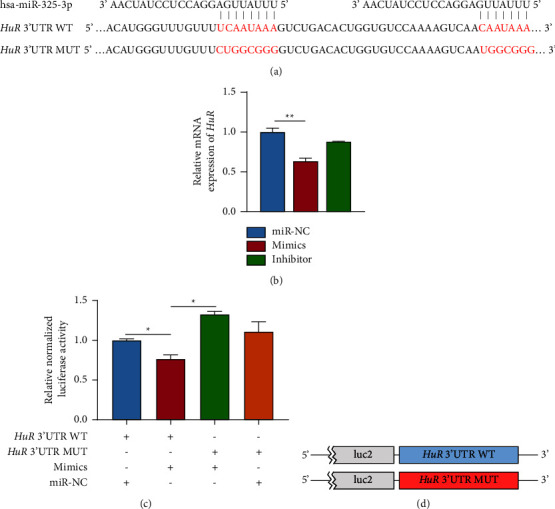
Overexpression of *miR*-*325*-*3p* reduced expression levels of *HuR* in SGC-7901 and HGC-27 cells. (a) Schematic diagram of potential binding sites and mutation sequence in *HuR* 3′UTR region. (b) Detecting relative expression levels of *HuR* mRNA by qRT-PCR. (c) Analyzing relative luciferase activity of each group by dual-luciferase reporter assays. (d) The schematic of two luciferase reporter plasmids (^*∗*^*P* < 0.05 and ^*∗∗*^*P* < 0.01, compared to siNC group).

Bioimformatic analysis uncovered that *HuR* mRNA 3'UTR region contained 2 potential *miR-325-3p* binding sites, which is highly conserved throughout the species. To further validate the interplay between *HuR* and *miR-325-3p*, both the original (*HuR* 3'UTR WT) and mutated (*HuR* 3'UTR MUT) sequences were cloned into the dual luciferase reporter (Figures 2(a) and 2(d)). Overexpression of *miR-325-3p* mimics led to a significant decrease in *HuR* mRNA levels in SGC-7901 cells (Figure 2(b)). Consistently, *miR-325-3p* mimics inhibited the relative luciferase activity of the *HuR* 3′UTR WT plasmid, whereas the corresponding luciferase activity of the *HuR* 3′UTR MUT plasmid was not affected (Figure 2(c)).

### 3.3. *miR-325-3p *Suppressed Proliferation and Enhanced Apoptosis in Gastric Cancer Cells

**Figure 3:**
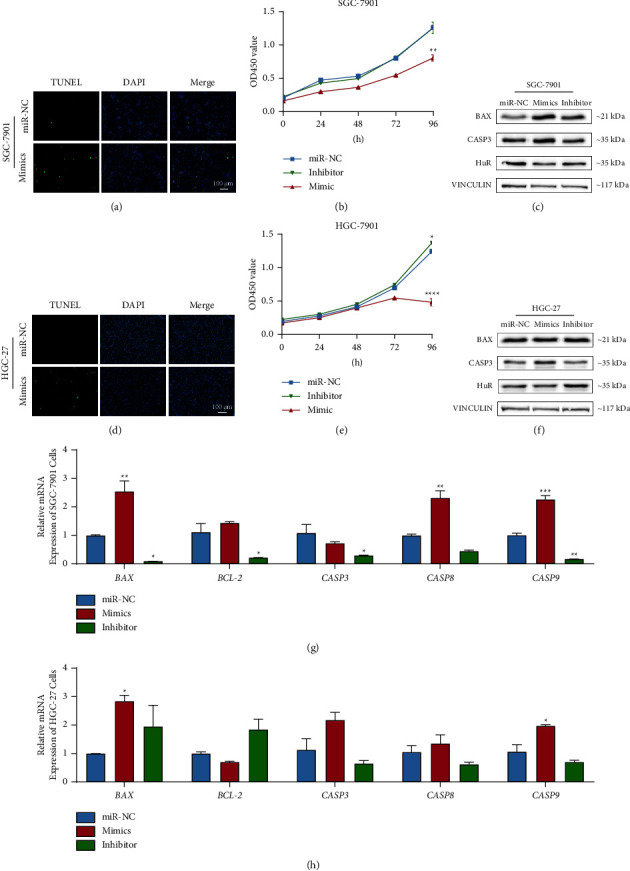
Overexpression of *miR-325-3p* reduced cell proliferation and increased apoptosis in SGC-7901 and HGC-27 cells. (a, d) Detecting the apoptosis ratio of SGC-7901 and HGC-27 cells by TUNEL assay. (b, e) Evaluating the cell proliferation of SGC-7901 and HGC-27 cells by CCK-8 assay within 96 hours. (c, f) Analyzing the apoptosis-related proteins by western boltting. (g, h) Analyzing the expression of apoptosis-related genes by qRT-PCR (^*∗*^*P* < 0.05, ^*∗∗*^*P* < 0.01, ^*∗∗∗*^*P* < 0.001, and ^*∗∗∗∗*^*P* < 0.0001, compared to siNC group).

After confirming the regulatory role of *miR-325-3p* in HuR expression by binding its 3′UTR in gastric cancer cells, the apoptotic and proliferative abilities of cells transfected with *miR-325-3p* mimics were evaluated. The group transfected with *miR-325-3p* mimics displayed increased apoptotic cells compared to the miR-NC group (Figures 3(a) and 3(d)). In terms of cell proliferation, cells transfected with *miR-325-3p* mimics exhibited a lower proliferative rate compared to both the miR-NC and inhibitor groups. However, there was no significant difference in proliferation rate between the miR-NC and inhibitor groups (Figures 3(b) and 3(e)). Overexpression of *miR-325-3p* mimics resulted in reduced *HuR* expression and increased CASP3 expression in both SGC-7901 and HGC-27 cells (Figures 3(c) and 3(f)). The gene expression associated with cell apoptosis resembled that when HuR was silenced (Figures 3(g) and 3(h)). These findings suggested that overexpression of *miR-325-3p* in gastric cancer cells reduced HuR synthesis, leading to decreased proliferation and increased apoptosis of gastric cancer cells.

### 3.4. *miR-325-3p* Slowed In Vivo Tumour Growth of SGC-7901 Cells

**Figure 4:**
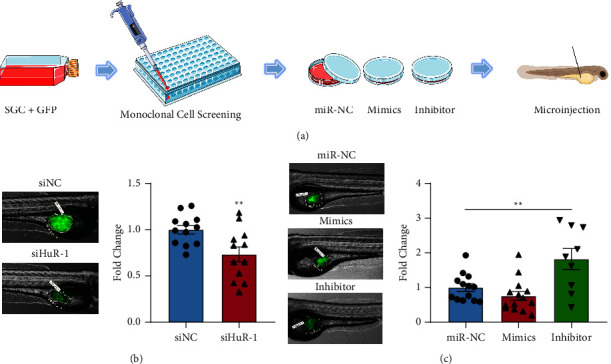
Overexpression of *miR-325-3p* lead to slowed growth of gastric tumors in zebrafish. (a) Schematic diagram of the fabrication of the SGC-7901-GFP monoclonal cell line and the microinjection of cells. (b) The proliferation of siRNA knockdown SGC-7901-GFP cells in zebrafish and the statistics of the fluorescence expression area (both groups *n* = 12). (c) After overexpression or inhibition of *miR-325-3p*, the proliferation of SGC-7901-GFP cells in zebrafish and the statistics of the fluorescence area were compared with those in miR-NC group (*n* = 13), mimics group (*n* = 14), and inhibitor group (*n* = 9).

To investigate the effects of *miR-325-3p* on gastric tumor growth, the SGC-7901 cells that were genetically modified to express green fluorescent protein (SGC-7901-GFP) via plasmid transfection and cell monoclonal screening were used. After transfection with either siHuR1 or *miR-325-3p* mimics, the corresponding SGC-7901-GFP cells were injected into larval zebrafish using microinjection (Figure 4(a)). The results indicated that the fluorescence expression area was lower in the HuR knockdown group and the *miR-325-3p* mimics group than that in the respective control group. Furthermore, the fluorescence expression area was significantly higher in the *miR-325-3p* inhibitor group than in the control group (Figures 4(b) and 4(c)). In summary, *miR-325-3p* may suppress the growth of gastric cancer cells by blockng the expression of HuR in zebrafish.

## 4. Discussion

Along with the growing number of studies examining the modulation of HuR function as a therapeutic target for anticancer drugs, the role of HuR in tumorigenesis and development has garnered increased recognition. Additionally, several studies have suggested that inhibiting HuR may prove to be a promising target for tumor therapy [31]. In this study, two *miR-325-3p* binding sites was identified at the 3′UTR of HuR through bioinformatics analysis. Furthermore, knockdown of HuR or overexpression of *miR-325-3p* led to increased apoptosis and decreased proliferation in both SGC-7901 and HGC-27 cells, as evidenced by CCK-8 and TUNEL experiments. Results of the TUNEL apoptosis detection experiments were consistent with the expression of apoptosis-related genes and proteins. Dual-Luciferase reporter assay additionally demonstrated that *miR-325-3p* could regulate its expression by binding to the 3′UTR of *HuR*. Finally, the effects of *miR-325-3p* on tumor growth were confirmed by *in vivo* zebrafish experiments. The results demonstrate that *miR-325-3p *regulates HuR expression to influence the proliferation of gastric cancer cells. This discovery offers a new strategy for developing HuR-targeting drugs for future cancer treatment..

HuR expression affects multiple tumor-related phenotypes [31]. Several experiments have demonstrated increased expression of HuR in various tumor cells, with limited exploration of HuR's role in gastric cancer [17]. In this study, siRNA was utilized to downregulate the expression of HuR in SGC-7901 and HGC-27 cells, resulting in decreased proliferation and increased apoptosis. This outcome is consistent with previous reports, providing additional evidence for the critical role played by HuR in tumor development. Importantly, it has been established that HuR also plays a significant role in preserving gastric cancer cells' division and proliferation.

The elevated expression of HuR has a significant impact on the prognosis of diverse diseases. Currently, three primary techniques exist to suppress HuR: (1) inhibiting the expression of HuR by miRNA or small molecule RNA [32, 33], (2) inhibiting the shuttling of HuR out of the nucleus by modified molecules [34], and (3) causing the loss of its function by using drugs that competitively bind to the specific locus of HuR [35]. Each of these methods has its corresponding advantages and disadvantages. The expression of *miR-325-3p* is decreased in gastric cancer patients, indicating it may regulate cisplatin resistance in this type of cancer [23]. *In vitro* experimental results and sequence comparison analysis revealed that *miR-325-3p* may reduce the expression of HuR by binding to the 3′UTR of *HuR*, which is consistent with classic miRNA regulation. 

Subsequent studies showed that a elevated expression of *miR-325-3p* mimics could inhibit the proliferation of gastric cancer cells. The inhibition on cell proliferation by overexpression of *miR-325-3p* mimics appeared to be gentler than the direct knockdown of HuR. Similarly, for apoptosis induction, overexpression of *miR-325-3p* mimics showed similar yet distinct results compared to HuR knockdown. TUNEL experiment demonstrated that higher *miR-325-3p* expression could effectively enhance gastric cancer cell apoptosis compared to the control group. There have been previous clinical trials that evaluated miRNAs could be used as pharmaceutical agents [22].This study provides a theoretical basis for potentially utilizing *miR-325-3p* as a therapeutic intervention for gastric cancer.

Zebrafish (*Danio rerio*) share a remarkable levle of physiological and genetic similarity with mammals. The zebrafish embryo is a promising model for xenograft tumors because of its transparency, which makes it convenient to observe and study tumor development and growth *in vivo* [36]. The experiment revealed that inhibiting *miR-325-3p* led to a significant increase in tumor volume in zebrafish compared to the control group. This finding might indicate that in animals, gastric cancer growth is more sensitive to *miR-325-3p* suppression.

Regarding *miR-325-3p* as a potential drug target, further investigation is required into its mechanistic and functional characterization. This study is limited by a low percentage of HuR knockdown by *miR-325-3p*, making it impossible to exclude the possibility that these two factors participate synergistically in the development of gastric cancer.

## 5. Conclusions

*miR-325-3p* inhibited the proliferation and promoted apoptosis of gastric cancer cells by binding to *HuR *3'UTR and suppress its expression. These results offer valuable insights into the pathogenesis of gastric cancer and establish new potential targets for therapeutic intervention.

## Data Availability

All data generated or analyzed during the study are included within the article.

## References

[1] Deng W., Jin L., Zhuo H., Vasiliou V., Zhang Y. (2021). Alcohol consumption and risk of stomach cancer: a meta-analysis. *Chemico-Biological Interactions*.

[2] Jin G., Lv J., Yang M. (2020). Genetic risk, incident gastric cancer, and healthy lifestyle: a meta-analysis of genome-wide association studies and prospective cohort study. *The Lancet Oncology*.

[3] Sung H., Ferlay J., Siegel R. L. (2021). Global cancer statistics 2020: GLOBOCAN estimates of incidence and mortality worldwide for 36 cancers in 185 countries. *CA: A Cancer Journal for Clinicians*.

[4] Smyth E. C., Nilsson M., Grabsch H. I., van Grieken N. C. T., Lordick F. (2020). Gastric cancer. *The Lancet*.

[5] Zhong Q., Chen Q. Y., Li P. (2018). Prediction of conditional probability of survival after surgery for gastric cancer: a study based on eastern and western large data sets. *Surgery*.

[6] de Silanes I. L., Zhan M., Lal A., Yang X., Gorospe M. (2004). Identification of a target RNA motif for RNA-binding protein HuR. *Proceedings of the National Academy of Sciences of the United States of America*.

[7] Liu Y., Chen X., Cheng R. (2018). The Jun/miR-22/HuR regulatory axis contributes to tumourigenesis in colorectal cancer. *Molecular Cancer*.

[8] Abdelmohsen K., Srikantan S., Kuwano Y., Gorospe M. (2008). miR-519 reduces cell proliferation by lowering RNA-binding protein HuR levels. *Proceedings of the National Academy of Sciences of the United States of America*.

[9] Chen Y., Yang F., Fang E. (2019). Circular RNA circAGO2 drives cancer progression through facilitating HuR-repressed functions of AGO2-miRNA complexes. *Cell Death & Differentiation*.

[10] Yang F., Hu A., Li D. (2019). Circ-HuR suppresses HuR expression and gastric cancer progression by inhibiting CNBP transactivation. *Molecular Cancer*.

[11] Li Q., Tong D., Guo C. (2020). MicroRNA-145 suppresses gastric cancer progression by targeting Hu-antigen R. *American Journal of Physiology-Cell Physiology*.

[12] Goutas D., Pergaris A., Giaginis C., Theocharis S. (2022). HuR as therapeutic target in cancer: what the future holds. *Current Medicinal Chemistry*.

[13] Zhang Z., Huang A., Zhang A., Zhou C. (2017). HuR promotes breast cancer cell proliferation and survival via binding to CDK3 mRNA. *Biomedicine & Pharmacotherapy*.

[14] Ahmed R., Muralidharan R., Srivastava A. (2021). Molecular targeting of HuR oncoprotein suppresses MITF and induces apoptosis in melanoma cells. *Cancers*.

[15] Kakuguchi W., Kitamura T., Kuroshima T. (2010). HuR knockdown changes the oncogenic potential of oral cancer cells. *Molecular Cancer Research*.

[16] Giammanco A., Blanc V., Montenegro G. (2014). Intestinal epithelial HuR modulates distinct pathways of proliferation and apoptosis and attenuates small intestinal and colonic tumor development. *Cancer Research*.

[17] Assoni G., La Pietra V., Digilio R. (2022). HuR-targeted agents: an insight into medicinal chemistry, biophysical, computational studies and pharmacological effects on cancer models. *Advanced Drug Delivery Reviews*.

[18] Filippova N., Yang X., Ananthan S. (2021). Targeting the HuR oncogenic role with a new class of cytoplasmic dimerization inhibitors. *Cancer Research*.

[19] Singh G., Storey K. B. (2021). MicroRNA cues from nature: a roadmap to decipher and combat challenges in human Health and disease?. *Cells*.

[20] Wang S., Wang Z., Wang Q., Cui Y., Luo S. (2019). Clinical significance of the expression of miRNA-21, miRNA-31 and miRNA-let7 in patients with lung cancer. *Saudi Journal of Biological Sciences*.

[21] Hill M., Tran N. (2021). miRNA interplay: mechanisms and consequences in cancer. *Disease Models & Mechanisms*.

[22] Rupaimoole R., Slack F. J. (2017). MicroRNA therapeutics: towards a new era for the management of cancer and other diseases. *Nature Reviews Drug Discovery*.

[23] Sun T., Li K., Zhu K., Yan R., Dang C., Yuan D. (2020). SNHG6 interacted with miR-325-3p to regulate cisplatin resistance of gastric cancer by targeting GITR&lt. *OncoTargets and Therapy*.

[24] Yao S., Zhao T., Jin H. (2015). Expression of MicroRNA-325-3p and its potential functions by targeting HMGB1 in non-small cell lung cancer. *Biomedicine & Pharmacotherapy*.

[25] Gan H., Lin L., Hu N. (2019). KIF2C exerts an oncogenic role in nonsmall cell lung cancer and is negatively regulated by miR-325-3p. *Cell Biochemistry and Function*.

[26] Li L., Ji Y., Chen Y. C., Zhen Z. J. (2021). MiR-325-3p mediate the CXCL17/CXCR8 axis to regulate angiogenesis in hepatocellular carcinoma. *Cytokine*.

[27] Westerfield M. (2007). The zebrafish book: a guide for the laboratory use of zebrafish (*Danio rerio*). *The Zebrafish Book: A Guide for the Laboratory Use of Zebrafish (Danio rerio)*.

[28] Wu W., Xu S., Guan K. (2020). 5-FU blocks shuttling of HuR mediated by PKC*δ* in gastric cancer cells. *Translational Cancer Research*.

[29] Lv J., Zhang S., Liu Y. (2021). NR2F1-AS1/miR-190a/PHLDB2 induces the epithelial-mesenchymal transformation process in gastric cancer by promoting phosphorylation of AKT3. *Frontiers in Cell and Developmental Biology*.

[30] Wu J. Q., Zhai J., Li C. Y. (2017). Patient-derived xenograft in zebrafish embryos: a new platform for translational research in gastric cancer. *Journal of Experimental & Clinical Cancer Research*.

[31] Wu X., Xu L. (2022). The RNA-binding protein HuR in human cancer: a friend or foe?. *Advanced Drug Delivery Reviews*.

[32] Wu X., Gardashova G., Lan L. (2020). Targeting the interaction between RNA-binding protein HuR and FOXQ1 suppresses breast cancer invasion and metastasis. *Communications Biology*.

[33] Lal S., Cheung E. C., Zarei M. (2017). CRISPR knockout of the HuR gene causes a xenograft lethal phenotype. *Molecular Cancer Research*.

[34] Meisner N. C., Hintersteiner M., Mueller K. (2007). Identification and mechanistic characterization of low-molecular-weight inhibitors for HuR. *Nature Chemical Biology*.

[35] Manzoni L., Zucal C., Maio D. D. (2018). Interfering with HuR-RNA interaction: design, synthesis and biological characterization of tanshinone mimics as novel, effective HuR inhibitors. *Journal of Medicinal Chemistry*.

[36] Snaar-Jagalska B. E., Zf-Cancer (2009). ZF-CANCER: developing high-throughput bioassays for human cancers in zebrafish. *Zebrafish*.

